# Implication of single nucleotide polymorphisms in *Interleukin-10* gene (*rs1800896* and *rs1800872*) with severity of COVID-19

**DOI:** 10.1186/s43042-022-00344-3

**Published:** 2022-10-01

**Authors:** Saliha Rizvi, S. Mohd.Shiraz Rizvi, Syed Tasleem Raza, Mohd. Abbas, Kaynat Fatima, Zeashan H. Zaidi, Farzana Mahdi

**Affiliations:** 1 Department of Biotechnology, Era’s Lucknow Medical College and Hospital, Era University, Lucknow, Uttar Pradesh 226003 India; 2 Department of Biochemistry, Era’s Lucknow Medical College and Hospital, Lucknow, Uttar Pradesh 226003 India; 3 Department of Personalized and Molecular Medicine, Era University, Lucknow, Uttar Pradesh 226003 India; 4 Department of Community Medicine, Era’s Lucknow Medical College and Hospital, Lucknow, Uttar Pradesh 226003 India

**Keywords:** COVID-19, Cytokines, Genotyping, *IL-10*, SARS-CoV-2

## Abstract

**Background:**

Coronavirus disease 2019 (COVID-19) is an ongoing pandemic which has emerged as a new challenge for the medical sciences. Severity of COVID-19 is mostly determined with overexpressed proinflammatory cytokines eventually leading to endothelial dysfunction causing vital organ injury, especially in the lungs. It has been postulated that various genetic mutations might be associated with an increased risk of disease severity in COVID-19. This study was thus carried out to determine the association of *rs1800896 and rs1800872* genetic polymorphism in IL-10 gene in determining COVID-19 severity.

**Methods:**

The study included 160 RT-PCR confirmed COVID-19 patients with mild (*n* = 85) and severe (*n* = 75) conditions. All subjects were genotyped for *Interleukin-10* (*rs1800896 and rs1800872)* gene polymorphisms using PCR–RFLP technique followed by statistical analysis.

**Results:**

This study found a significant gender and age-based discrepancy in COVID-19 severity with 1.85-and 3.81-fold increased risk of COVID-19 in males of mild and severe groups as compared to females (*p* = 0.046 and *p* < 0.001) and 4.35-fold high risk in subjects ≥ 50 (*p* < 0.001). Genotyping analysis showed that *IL-10 (rs1800872)* gene polymorphism was strongly associated with COVID-19 severity (*p* = 0.01) whereas, *IL-10 rs1800896* polymorphism was not found to confer the risk of COVID-19 severity in our population.

**Conclusion:**

In this regard, the present study provided an evidence that *IL-10 (rs1800872)* gene polymorphism is strongly associated with COVID-19 severity and CC genotype confer a protective role in preventing severe disease progression. More detailed studies with a larger sample size on the genetic variations are required to establish the role of studied *IL-10* gene polymorphisms with COVID-19 severity.

## Background

Coronavirus disease 2019 (COVID-19) is an ongoing pandemic which has emerged as a new challenge for the medical sciences. It is a highly contagious disease, which occurs in humans after infection with severe acute respiratory syndrome coronavirus 2 (SARS-CoV-2). The Global reports till May 31, 2021, had confirmed more than 170 million cases of COVID-19 (26.9 million cases reported in India) with more than 3.54 million confirmed deaths, making it one of the deadliest pandemics in history [1, 2].

During the course of this pandemic outspread it had been observed that COVID-19 infection and severity is highly heterogeneous; where majority of COVID-19 cases may be either asymptomatic or have only mild symptoms, while some of the patients develop serious health outcomes resulting from various immunologic complications, such as macrophage activation, cytokine storm and acute respiratory distress syndrome [3]. The persistence of slower clearance rate of SARS-CoV-2 infection is subject to factors such as genetic, viral features, lower levels of interferons, macrophage activation, cytokine storm and probable other unknown mechanisms which might lead to disease severity and related complications [3]. Severity of COVID-19 is mostly determined with overexpressed proinflammatory cytokines eventually leading to endothelial dysfunction which could further cause vital organ injury, especially in the lungs [4]. Previous studies on genetic polymorphism have reported an association of single nucleotide polymorphism in cytokine genes with high risk of sepsis and septic shock [5]. Although, cytokine storm in COVID-19 patients is similar to that previously seen in SARS patients infected by SARS-CoV, but a distinctive characteristic of COVID-19 cytokine storm is a drastic increase in interleukin 10 (IL-10) serum levels in severe patients [6–9]. Furthermore, IL-10 is elevated earlier than IL-6 in COVID-19 patients [7]. The serum levels of IL-10 strongly correlated with those of IL-6 and other inflammatory markers such as C-reactive protein [8].

Interindividual variations in IL-10 production are genetically contributed by polymorphisms within the promoter region. An earlier study on *IL-10* genetic polymorphism had reported an association of rs1800896 (− 1082 G/A) SNP with higher IL-10 serum levels and an increased risk of pneumonia severity [10]. This gene is an anti-inflammatory cytokine that prevents the protective immune response to pathogens by blocking the production of proinflammatory cytokines [11]. It is present on long arm of chromosome 1 (1q31–1q32) locus and produced by both myeloid cells and T cells. The polymorphism r*s1800896* occurs within a putative Ets (E26 transformation-specific) transcription factor-binding site and may affect the binding of this transcriptional factor leading to altered levels of IL-10 cytokine [12, 13]. On the other hand, 592C > A polymorphism (rs1800872) of *IL-10* gene leads to a significant loss in the negative promoter function, thus altering the IL-10 transcription and mRNA expression [14]. This study was thus carried out to determine the association of *rs1800896 and* rs1800872 genetic polymorphism in *IL-10* gene since we hypothesize that these polymorphisms might play an important role in determining COVID-19 severity.

## Methods

### Study participants

This study included a total of 160 patients diagnosed with COVID-19 and admitted in Era’s Lucknow Medical College and Hospital, Lucknow. COVID-19 was confirmed in patients by testing their naso-pharyngeal swab samples by SARS-CoV-2 RNA real-time reverse transcription PCR (RT-PCR) test. The COVID-19 patients were divided into two groups mild (*n* = 85) and severe (*n* = 75). These diagnostic criteria were based on the recommendations by the Indian Council of Medical Research (ICMR), New Delhi, India. Patients with respiratory rate less than 24 per minute and SpO_2_ > 94% on room air were considered as mild patient while patients with respiratory rate more than 30 per minute OR SpO_2_ < 90% on room air with pneumonia were categorized into severe patients.

An informed consent was taken from all patients prior to sample collection and the study was approved by Institutional Ethical Committee of Era’s Lucknow Medical College and Hospital, Lucknow. All procedures in this study involving human participants were performed in accordance with the ethical standards of Era’s Lucknow Medical College and Hospital, Lucknow. Patients lacking blood test and medical history, less than 20 years of age, pregnant woman, immunocompromised patients and those undergoing dialysis were excluded from the study.

### Data collection and blood sampling

The clinical and biochemical details of patients were collected as demographic data, clinical history of diabetes and hypertension, family history, associated complications and other clinical data from hospital’s electronic medical records under supervision of an expert clinician (Table 1). After an informed consent from patient, 2 mL of peripheral blood sample were collected from all patients in vials coated with ethylene diamine tetra acetic acid (EDTA) and stored at − 20 °C for genotyping.

**Table 1:** Demographic and biochemical characteristics of the study participants COVID-19 patients

Demographic/biochemical parameters	Mild COVID-19 patients (*n* = 85)		Severe COVID-19 patients (*n* = 75)		Unpaired *t* test	
	Mean	SD	Mean	SD	*t *value	*p* value
Sex (M/F)	49/36	–	51/24	–	–	–
Age	43.9	16.5	50.2	14	2.612	**0.01**
BMI	26.7	8.2	28.81	12.32	1.26	0.210377
Urea	30.76	21.65	59.58	53.29	− 4.51	**< 0.001**
Creatinine	1.23	2.26	1.65	2.42	− 1.14	0.256
Sodium	138.52	3.55	139.21	5.25	− 0.97	0.335
Potassium	4.11	0.37	4.10	0.56	0.15	0.878
Bilirubin	0.61	0.26	1.02	2.80	− 1.31	0.191
SGPT	49.62	55.41	79.13	180.44	− 1.41	0.160
SGOT	51.24	36.39	81.82	149.90	− 1.79	0.075
Alkaline phosphatase	101.94	39.36	110.53	48.99	− 1.22	0.223
Calcium	8.81	0.60	8.65	0.66	1.68	0.094
Total Protein	7.07	0.66	6.34	0.90	5.89	**< 0.001**
Albumin	3.62	0.61	2.94	0.55	7.38	**< 0.001**
Hb	12.47	2.06	11.71	2.35	2.17	**0.032**
Total leucocyte count	7484.34	3469.89	12,848.00	7561.50	− 5.82	**< 0.001**
Neutrophils	69.20	12.46	85.07	10.74	− 8.53	**< 0.001**
Lymphocytes	25.05	11.65	11.29	9.39	8.11	**< 0.001**
Eosinophils	2.66	3.00	1.48	1.14	3.21	**0.002**
Monocytes	3.08	1.30	2.17	1.56	4.00	**< 0.001**
Platelets	2.15	0.87	2.22	1.13	− 0.46	0.646

Statistically signficant values are shown in bold

*n* number, % percentage, *SD* standard deviation, *SGPT* serum glutamic pyruvic transaminase, *SGOT* serum glutamic oxaloacetic transaminase, *Hb* hemoglobin

Significant association (*p* < 0.05)

### Genotyping

#### DNA isolation

Genomic DNA was extracted from peripheral blood samples by using a commercially available kit (Nucleospin blood, India) and the quality/quantity of DNA was assessed using a nanodrop spectrophotometer (Thermo Scientific, India) and checked on 1% agarose gel.

#### Polymerase chain reaction-restriction fragment length polymorphism (PCR–RFLP)

The genotyping in the present study was confirmed via polymerase chain reaction (PCR)-restriction fragment length polymorphism (RFLP) analysis.

#### Interleukin-10 gene (rs1800896)

Forward: 5′-CTCGCCGCAACCCAACTGGC -3′

Reverse: 5′-TCTTACCTATCCCTACTTCC -3′

The PCR reaction was performed in a 20-µL reaction volume containing 50 ng genomic DNA, 10 mL Taq PCR mix (Takara containing 1 mmol/L MgCl_2_, 100 mmmol/L deoxynucleotide triphosphate (dNTP), 0.5 U Taq polymerase and 10 pmol primers. PCR conditions were as follows: initial denaturation step at 95 °C for 5 min, 35 cycles of 95 °C for 30 s, 58.6 °C for 40 s, 72 °C for 60 s and final extension at 72 °C for 5 min. Cycling conditions were standardized on conventional PCR machine (BIO-RAD, India). After successful amplification, the 134 bp PCR products were digested with 2.5U of MnlI (New England Biolabs, USA) at 37 °C for overnight. The digested products were run on 2.5% agarose gel checked and visualized by gel documentation system (BIO-RAD, India). The undigested PCR product with 134 bp represented T allele. The presence of G allele was confirmed by visualizing two fragments of digested PCR product with 101 bp and 33 bp.

#### Interleukin-10 gene (rs1800872)

Forward: 5′-CTCGCCGCAACCCAACTGGC -3′

Reverse: 5′-TCTTACCTATCCCTACTTCC -3′

The PCR reaction was performed in a 20-µL reaction volume containing 50 ng genomic DNA, 10 mL Taq PCR mix (Takara containing 1 mmol/L MgCl_2_, 100 mmmol/L deoxynucleotide triphosphate (dNTP), 0.5 U Taq polymerase and 10 pmol primers. PCR conditions were as follows: initial denaturation step at 95 °C for 5 min; 35 cycles of 95 °C for 30 s, 51.3 °C for 30 s and 72 °C for 45 s, which was followed by a final extension step of 72 °C for 10 min. After successful amplification, the 482 bp PCR products were digested with 5U of AfaI (New England Biolabs, USA) at 37 °C for overnight. The digested products were run on 3% agarose gel checked and visualized by gel documentation system (BIO-RAD, India). The undigested PCR product with 482 bp represented C allele. The presence of G allele was confirmed by visualizing two fragments of 258 bp and 224 bp.

### Statistical analysis

All the statistical analyses were performed with SPSS (Statistical Package for the Social Sciences) version 21 software (IBM Corp., Chicago, Illinois, USA). Variables were tested for normality using the Kolmogorov–Smirnov test. Variables with a normal distribution were expressed as means ± standard deviation while variables with a non-normal distribution were expressed as medians and interquartile ranges and were log transformed before statistical analysis. The genotyping data were compared between cases and controls using Chi-square test. The unpaired t-test was used to compare the biochemical parameters of the two study groups. Other variables were compared using z-test for normally distributed variables. P-values ≤ 0.05 were considered as significant. Odds ratios (OR) and 95% confidence intervals (CI) were calculated to test the relative risk for association.

## Results

### Clinical characteristics of patients

A total of 160 patients diagnosed with COVID-19 were enrolled in this study, including 85 patients with mild and 75 patients with severe disease symptoms. The mean ages were 45.93 ± 16.5 years in the mild group and 58.18 ± 14.02 years in the severe group. The patients in the mild group included 36 females and 49 males while in the severe group had 25 females and 50 males. This study observed probable gendered implications of COVID-19 where frequency of males was significantly higher in both mild and severe patients (57.65% and 68%) as compared to females (42.35% and 32%). There was a significant 1.85-fold (*p* = 0.046) and 3.81-fold (*p* < 0.001) increased risk of COVID-19 in males of mild and severe groups as compared to females (Table 2). The study also reported a 4.35-fold high risk of disease severity in subjects ≥ 50 years of age while the disease intensity was significantly mild in subjects < 50 years of age (*p* < 0.001) as shown in Table 3. A total of 7 deaths (9.33%) were observed from 75 severe patients (> 55 years of age) including 4 male and 3 female patients.

**Table 2:** Gender-based comparison of COVID-19 severity

Gender	Female		Males		OR (95% CI)	*p* value
	*N*	%	*N*	%		
Mild (*n* = 85)	36	42.35	49	57.65	1.85 (1.01–3.40)	**0.046**
Severe (*n* = 75)	24	32	51	68	3.81 (2.01–7.24)	**< 0.001**

Statistically signficant values are shown in bold

*N* number, % percentage

Significant association (*p* < 0.05)

**Table 3:** Age-based comparison of COVID-19 severity

Age	Mild (*n* = 85)		Severe (*n* = 75)		OR (95% CI)	*p* value
	*N*	%	*N*	%		
< 50 years	46	54.1	16	21	0.23(0.11–0.46)	**< 0.001**
≥ 50 years	39	45.9	59	79	4.35(2.16–8.74)	**< 0.001**

Statistically signficant values are shown in bold

*N* number, % percentage

Significant association (*p* < 0.05)

Comparisons of biochemical data between mild and severe COVID-19 patients is shown in Table 1. The patients of severe group had significantly higher value of blood urea, TLC and Neutrophil count and a lower value of total protein, albumin, Hb and lymphocytes, eosinophils, monocytes count as compared to patients in the mild group (*p* < 0.05).

### Genotyping analysis

#### IL-10 (rs1800896) gene polymorphism

**Fig. 1:**
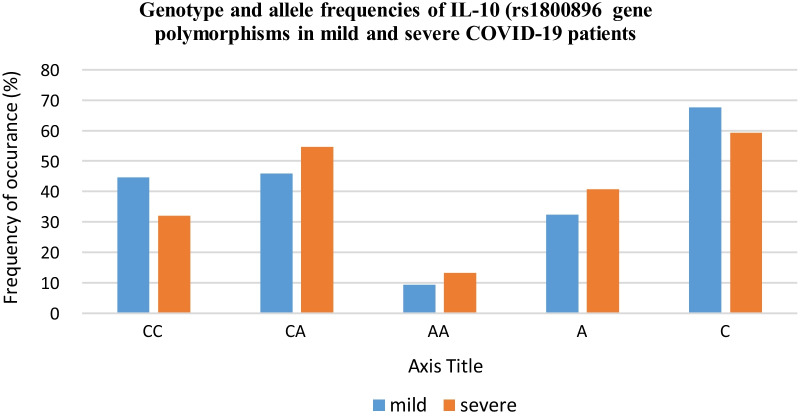
Graphical representation of genotype and allele frequencies of *IL-10 rs1800872* gene polymorphisms in COVID-19 patients

The frequency of AA and GG genotypes was higher among severe patients than in mild (65.3% vs. 52.9%; 4% vs. 2.4%, respectively) that showed the corresponding marginal increased risk of severity with homozygous genotypes (Fig. 1). Individuals with AA and GG genotypes showed a 1.68 and 1.73‐folds higher risk of severity due to COVID‐19 infection. However, in case of AG genotype, its frequency of occurrence was lower in severe cases (30.7%) as compared to mild cases (44.7%). However, the results failed to show any significant association of *IL-10 (rs1800896)* genotypic variants with COVID-19 severity (*p* > 0.05).

#### IL-10 (rs1800872) gene polymorphism

**Fig. 2:**
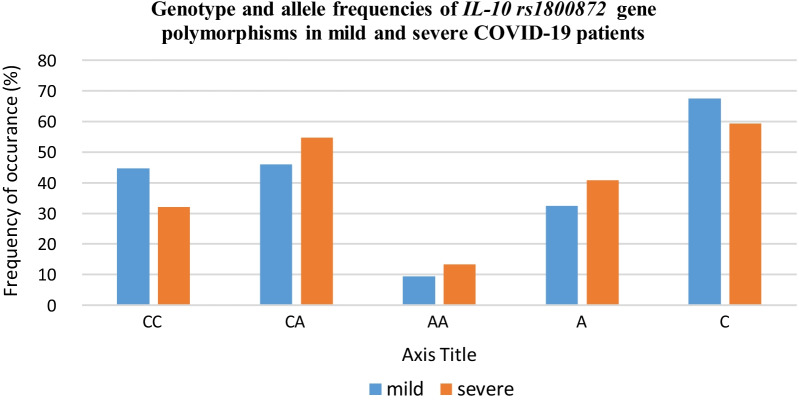
Graphical representation of genotype and allele frequencies of *IL-10 rs1800896* gene polymorphisms in COVID-19 patients

The frequency of CA and AA genotypes and A allele was higher among severe patients than in mild (54.7% vs. 49.5%; 13.3% vs. 9.4%, and 40.7% vs. 32.4%, respectively) that showed the corresponding marginal increased risk of severity with homozygous genotypes (Fig. 2). Individuals with CA, AA genotypes and A allele showed a 1.42, 1.48 and 1.43‐folds higher risk of severity due to COVID‐19 infection. However, the results failed to show any significant association (*p* > 0.05). On the other hand, the frequency of occurrence of CC genotype was found to be significantly lower in severe group (32%) as compared to mild group (44.7%) suggesting its protective role in decreasing the risk of COVID-19 severity (*p* = 0.01).

The distribution of *IL-10 (rs1800896 and rs1800872)* genotypes/alleles in mild and severe patients are shown in Table 4.

**Table 4:** Distribution of genotype and allele frequencies of IL-10 (rs1800896 and rs1800872) gene polymorphisms in mild and severe COVID-19 patients

	Mild (*n* = 85)		Severe (*n* = 75)		Statistical analysis			
Genotypes/alleles	*N*	%	*N*	%	Chi-sq	*p* value	OR	95% CI
*IL-10 (rs1800896)*								
AA	45	52.9	49	65.3	2.52	0.112	1.68	(0.88–3.17)
AG	38	44.7	23	30.7	3.33	0.068	0.55	(0.29–1.05)
GG	2	2.4	3	4.0	0.36	0.550	1.73	(0.28–10.64)
G	42	24.7	29	19.3	1.33	0.248	0.73	(0.43–1.25)
A	128	75.3	121	80.7	1.33	0.248	1.37	(0.80–2.34)
*IL-10 (rs1800872*)								
CC	38	44.7	24	32.0	2.71	**0.010**	0.58	(0.30–1.11)
CA	39	45.9	41	54.7	1.23	0.267	1.42	(0.76–2.65)
AA	8	9.4	10	13.3	0.61	0.433	1.48	(0.55–3.97)
A	55	32.4	61	40.7	2.38	0.123	1.43	(0.91–2.26)
C	115	67.6	89	59.3	2.38	0.123	0.70	(0.44–1.10)

Statistically signficant values are shown in bold

## Discussion

The COVID‐19 pathogenesis harbors an effective inflammatory response, which activates a group of inflammatory mediators including interleukins. Hyperimmune activation during the course of the disease leads to excessive production of pro‐inflammatory cytokines resulting in “**cytokine storm**” that triggers disease severity and acute organ injuries. IL-10 is an inflammatory cytokine whose concentration was found to increase in patients that develop severe/critically ill condition post COVID-19 infection [15]. A recent meta-analysis from 18 clinical studies identified *IL-10* as a covariate that accurately predicted disease severity in COVID-19 patients [16]. A study conducted on COVID-19 patients, showed a significant increase in serum IL-10 levels in critical group (*n* = 17) than in moderate (*n* = 42) and severe (*n* = 43) group [7]. As various approaches are being taken to uncover biological networks underlying host–pathogen (SARS-CoV-2) interactions and genetic basis of disease severity, we had studied *rs1800896 and rs1800872* polymorphisms of *IL-10* gene since it was found to alter serum levels of IL-10. The G to A polymorphism at − 1082 position regulates transcription of *IL‐10* gene. High levels of IL‐10 were recorded in severe COVID‐19 patients and found to be associated with the compensatory anti‐inflammatory response syndrome that may be responsible for a greater number of secondary infections (50%) and sepsis (100%) reported in survivors [17]. A study reported that individuals carrying GG genotype have increased levels of IL‐10 transcription and higher concentrations of circulating IL‐10 as compared to AA genotype carriers [18]. In the present study, in *IL-10 (rs1800896)* polymorphism it was observed that the heterozygous AG genotype was nearly significantly associated with decreased risk of disease severity (*p* = 0.068) whereas, no significant association of AA and AG genotype was observed with severity of COVID-19 in our study (*p* > 0.05). A study conducted on 23 countries reported a significant positive correlation between the frequency of AG genotype of *rs1800896* and prevalence of COVID-19 [19]. Population diversities of IL‐10 gene polymorphisms at *rs1800896* locus showed that the populations of China, Mexico, Tunisia, and Japan frequently have the AA genotype while the populations of India, Iran, Spain, Netherland, Finland, Brazil, Czechia, Poland, Germany, Norway, and the UK frequently have the AG genotype. The frequency of GG genotype of *rs1800896* polymorphism was the highest only among the Italian population [19].

On comparing allele frequencies, the prevalence of A allele was found to be higher in severe (80.7%) as compared to mild cases (75.3%) while, G allele was more frequently observed in mild (24.7%) as compared to severe patients (19.3%) in our study. However, these differences were not found to be statistically significant. Recently, a study on Chinese Han patients also reported that A allele of rs1800896 in *IL-10* gene could act as a risk indicator in pneumonia‐induced sepsis signifying A allele as a risk allele for pulmonary infections [20].

Population diversities of *IL‐10* gene polymorphisms at *rs1800896* locus showed that the populations of China, Mexico, Tunisia, and Japan frequently have AA genotype while the populations of India, Iran, Spain, Netherland, Finland, Brazil, Czechia, Poland, Germany, Norway, and UK have the AG genotype. The frequency of GG genotype of *IL-10* (*rs1800896)* polymorphism was the highest only among the Italian population [21].

In case of *IL-10 (*rs1800872) gene polymorphism, a significant protective role of CC genotype was observed in preventing COVID-19 severity among infected patients (*p* = 0.010). A study on Mexican population reported a similar finding where frequency of CC genotype was higher in mild (52.1%) as compared to severe COVID-19 patients (50.7%) but their results were not found to be significant [22]. The frequency of AA genotype was found to be 9.4% and 13.3% in our mild and severe cases respectively which is a bit higher when compared to Mexican COVID-19 patients (5.3% and 10.7%, respectively). However, comparison of other genotypes and allele between mild and severe patients did not reach a statistical difference in the present study and therefore do not support a role in disease severity. Studies from different ethnicities like Hong Kong and China discarded the association of this polymorphism with both susceptibility and severity for other viral infections like influenza A/H1N1pdm09 and for acute respiratory syndrome in Indian ethnic group [23, 24]. The opposite effect was reported in Mexican population, where *IL-10 (rs1800872)* was found to be associated with susceptibility to influenza, whereas *rs1800871* had no effect such effect [25]. In this type of studies, the contradictory results could be explained from immunogenetics and population genetics point of view considering the differential human immune response against viruses and by the genetic structure of populations.

On studying the effect of age on COVID-19 severity we found a highly significant association of age with COVID-19 severity where, people ≥ 50 years of age were found to be at risk of developing a severe form of COVID-19 (*p* < 0.001) as compared to patients ≤ 50 years of age. However, a recent meta-analysis conducted to study the effect of age on severe COVID-19 outcomes reported a rather weak influence of age on COVID-19 disease severity and death [26].

Gausman and Langer in 2020 studied sex and gender disparities in the COVID-19 patients suggesting the fact that immune response is a significant feature of sexual dimorphism and women show a stronger immune responses than males [27]. Thus on sex-based comparison of COVID-19 severity among both males and females a significantly strong association of COVID-19 risk was observed in males as compared to females in both mild and severe group in our study (as shown in Table 2) which is in agreement with the reporting from another study from China where men were found to be more predisposed to being affected by COVID-19 than women [28].

## Conclusion

The ongoing COVID-19 pandemic is a threat to mankind and it is very crucial to identify host genomic factors that increase susceptibility or resistance to the complications of the disease, so as to successfully translate these findings in a timely manner to improved patient care. In this regard, the present study provided an evidence that *IL-10 (rs1800872)* gene polymorphism is strongly associated with COVID-19 severity where, CC genotype confer a protective role in preventing severe disease progression. However, we observed no association of *IL-10 (rs1800896*) gene polymorphism with COVID-19 severity in our population. This study observed probable gendered implications of COVID-19 where frequency of males was significantly higher in both mild and severe patients as compared to females. The study also reported a 4.35-fold high risk of disease severity in subjects ≥ 50 years of age while the disease intensity was significantly mild in subjects < 50 years of age. More detailed and large sampled studies about the genetic variations in infected patients with different degrees of severity are needed to explain the underlying mechanism of different immune responses including the cytokine storm in COVID‐19 patients.

## Data Availability

Not applicable.
